# Diagnostic value of doppler ultrasound indices of maternal renal interlobar vasculature in the prediction of preeclampsia

**DOI:** 10.25122/jml-2019-0161

**Published:** 2021

**Authors:** Mohammad Gharib Salehi, Elham Shobeiri, Farhad Naleini, Mohammad Saied Bazargan

**Affiliations:** 1. Department of Radiology, School of Medicine, Kermanshah University of Medical Sciences, Kermanshah, Iran

**Keywords:** preeclampsia, pregnancy, Doppler ultrasound

## Abstract

We aimed to determine the diagnostic accuracy of maternal renal vasculature Doppler ultrasound indices in the prediction of preeclampsia. A total of 40 pregnant women with a gestational age of more than 20 weeks were included and followed. The pregnant women underwent a Doppler ultrasound examination to measure the resistance index (RI) of the interlobar arteries of right and left kidneys and the renal interlobar vein impedance index (RIVI). Of the included women, 15 patients developed preeclampsia based on clinical and laboratory criteria. The renal vascular Doppler ultrasound indices were compared between the two groups. Then, using the Receiver Operating Characteristic (ROC) analysis, the diagnostic accuracy of interlobar artery RI and RIVI were investigated in the prediction of preeclampsia occurrence. RIVI values of right and left kidneys were significantly higher in the preeclampsia group compared to the normal pregnancy group. The left kidney interlobar artery RI at a cut-point of 0.59 had a sensitivity of 100% and a specificity of 40% (area under curve=0.7, P-0.03) in the prediction of preeclampsia. The RIVI of the left kidney (adjusted odds ratio=17.14, 95% CI = 3.46 to 47.28) was statistically significant in predicting preeclampsia (P-0.006). We found that, besides other routine methods, using Doppler ultrasound and measuring RIVI can be reliable in the prediction of preeclampsia.

## Introduction

Preeclampsia is one of the most important and life-threatening conditions in pregnancy and. It is an unknown pregnancy complication in 3–7% of pregnancies. The pathophysiology of preeclampsia is not well known, but one of the most important factors is a systemic vascular dysfunction that causes local bleeding, necrosis and dysfunction with vascular and local hypoxia [1]. In addition, endothelial dysfunction can cause impaired vascular dysfunction of the placenta and hypertension [2]. Based on this evidence, measurements of maternal vasculature indices using Doppler ultrasound have been used to predict the occurrence of preeclampsia [3].

The kidneys regulate the blood pressure for a long time. In normal pregnancy, it is associated with a decrease in blood pressure and an increase in the renal plasma flow, and a glomerular filtration rate of about 30–40% [4]. The Doppler examination of the arterial tree involves measuring the resistance index (RI), which is defined as the systolic and diastolic velocity difference divided by systolic velocity. This index is, in fact, a measure of impedance that combines both low flow resistance and vascular adaptation [5]. The low flow resistance of the venous side of the arterial tree is usually negligible. Therefore, RI could be confusing, and the term “impedance index” is preferred regarding the venous side of the vasculature.

There is an increase in interstitial pressure in acute obstructive uropathy, and this is associated with a reduction in the intravenous impedance index [6]. If a change in the renal pressure natriuresis curve occurs, it can contribute to the pathophysiological mechanism of preeclampsia. Therefore, it may be used to predict renal impairment reduction in pregnant women with preeclampsia compared to normal pregnancies. Changes in the median pressure should change the intravenous impedance in the opposite direction that the kidney obstruction is found [7].

Regarding the evidence that dysfunctions in venous hemodynamics occur in preeclampsia [8, 9], it is beneficial to use non-invasive investigations such as Doppler ultrasound to measure renal vasculature tone and impedance in predicting preeclampsia. Some experts have proposed that an increase in renal interlobar vein impedance (RIVI) index occurs in preeclampsia [10]. However, there are authors who have not confirmed such findings [3]. Considering the contradictory results from the literature, this study aimed to evaluate the diagnostic value of renal Doppler indexes in the prediction of preeclampsia.

## Material and Methods

The study population comprised pregnant women referred to the Radiology Department for ultrasound evaluation of fetal health. The participants were recruited by convenience sampling. The inclusion criteria were the first pregnancy with a gestational age greater than 20 weeks based on the last menstrual period (LMP). Exclusion criteria were the history of diabetes mellitus (DM) or hypertension (HTN), the history of renal diseases known before pregnancy, and the presence of parenchymal kidney disease on gray-scale ultrasound performed before Doppler examination.

A preliminary study was initially conducted. Based on the results, a sample size of 40 subjects was calculated (P=0.97, 5% accuracy and a 95% confidence interval).

Preeclampsia was diagnosed by an obstetrician who followed the patients. Blood pressure measurements and proteinuria investigation were obtained. In the case of elevated blood pressure of greater than 140/90 mmHg, proteinuria of +1 and higher, the diagnosis of preeclampsia was made.

Doppler ultrasound examination included a review of renal parenchyma by removing any significant disturbances as well as a Doppler study of the interlobular vessels and the veins of both kidneys. The Doppler angle was kept at a minimum level by selecting the midpoint of the medullary vessel along with the possible rays. The impedance index was measured. In order to standardize the technique, a single radiologist performed all ultrasound examinations. All patients were physically and clinically examined prior to ultrasonography. Doppler ultrasound was performed by the Medison A30 model and the 4-9BE probe. The study variables included age, gestational age, body mass index (BMI), renal side, and Doppler ultrasound indices. The interlobar artery RI as well as RIVI were measured on both sides and documented.

### Statistical analysis

To compare the variables between normal pregnancy and preeclampsia groups, the Student t-test or Mann-Whitney U tests were used. To determine the optimal cut-off point and related sensitivity, specificity, positive predictive value (PPV), and negative predictive values (NPV) of the optimal cut-off points for RI and RIVI were calculated using the Receiver Operating Characteristic (ROC) curve analysis. A multivariate logistic regression method was used to determine the significant factors affecting the development of preeclampsia. Independent variables of this model included the interlobar artery RI and RIVI of right and left kidneys. The dependent variable was “normal pregnancy” or “preeclampsia”. The significance level was considered to be 0.05. The analyses were performed using the Statistical Package for the Social Sciences (SPSS) software (version 22.0).

## Results

Of 45 pregnant women included, 15 developed preeclampsia (33.3%) and 30 had a normal pregnancy. Table 1 presents a comparison of demographic and Doppler ultrasound characteristics between the two groups. As observed, right and left kidney RIVI values were significantly higher in the preeclampsia group compared to the normal pregnancy group.

**Table 1.** Comparison of demographic and Doppler ultrasound characteristics between preeclampsia and normal pregnancy.

	Normal pregnancy (n=30)	Preeclampsia (n=15)	P-value
**Maternal age, year**	27.6 (±2.74)	28.66 (±3.47)	0.26
**Gestational age, week**	34.66 (±2.27)	33.46 (±1.5)	0.068
**BMI, kg/m^2^**	30.79 (±3)	30.15 (±2.41)	0.47
**Right kidney RI**	0.59 (±0.032)	0.61 (±0.02)	0.065
**Left kidney RI**	0.6 (±0.032)	0.61 (±0.015)	0.044
**Right kidney RIVI**	0.32 (±0.039)	0.4 (±0.051)	<0.001
**Left kidney RIVI**	0.34 (±0.039)	0.45 (±0.048)	<0.001

BMI – body mass index; RI – resistance index; RIVI – renal interlobar vein impedance index. Data are presented as mean (± standard deviation).

### Diagnostic accuracy of interlobar artery RI for prediction of preeclampsia

**Figure 1.**
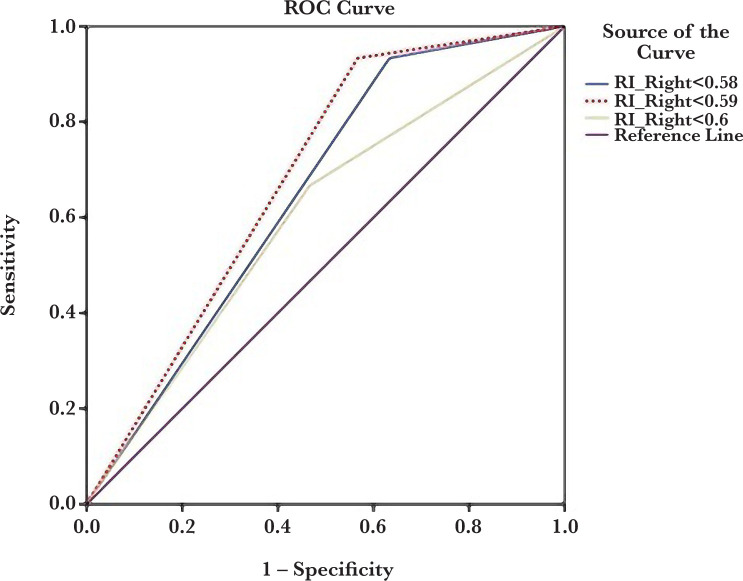
ROC curve analysis of the right kidney interlobar artery resistance index (RI) to predict preeclampsia at three different cut-points (0.58, 0.59, and 0.6).

**Figure 2.**
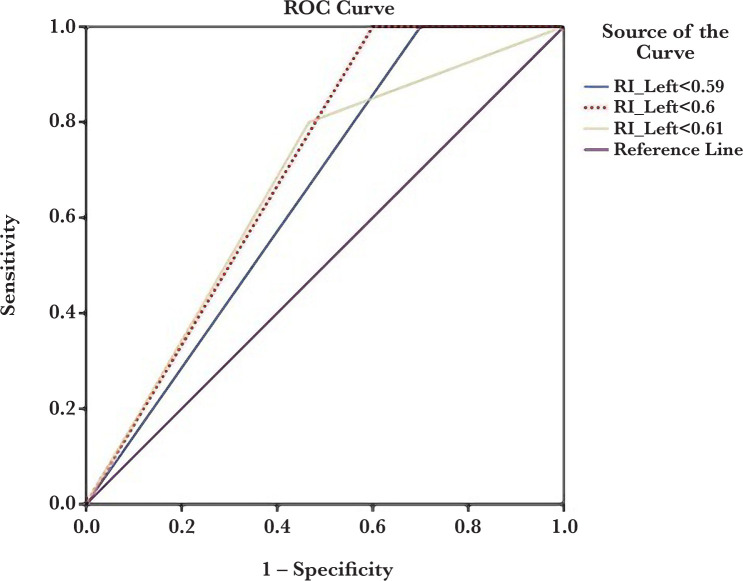
ROC curve analysis of the left kidney interlobar artery resistance index (RI) to predict preeclampsia at three different cut points (0.59, 0.6, and 0.61).

Table 2 presents the sensitivity, specificity, PPV, and NPV of the right and left kidney interlobar artery RI for preeclampsia prediction. The optimal cut-point for right and left kidney interlobar artery RI values were 0.59 and 0.6, respectively. As seen in Table 2, left kidney interlobar artery RI had better diagnostic accuracy than right kidney-related values in preeclampsia prediction. ROC curve analysis results for three cut-points are presented in Figures 1 and 2.

**Table 2.** Diagnostic accuracy of right and left kidney interlobar artery RI in the prediction of preeclampsia.

	Cut-point	Sensitivity	Specificity	PPV	NPV	Accuracy	AUC	P-value
**Right kidney**	0.59	93.3%	43.3%	45.1%	92.8%	60%	0.68	0.047
**Left kidney**	0.6	100%	40%	45.4%	100%	60%	0.7	0.03

AUC – area under curve; NPV – negative predictive value; PPV – positive predictive value.

### Diagnostic accuracy of renal interlobar vein impedance index for preeclampsia prediction

**Figure 3.**
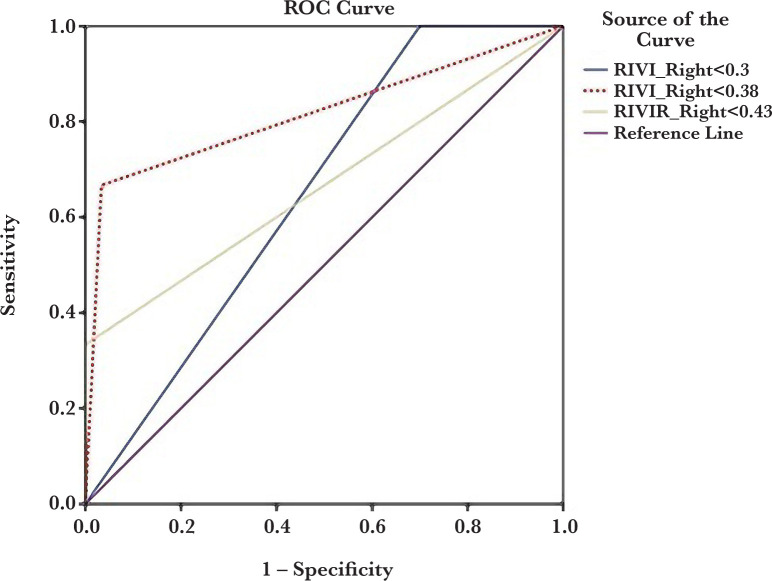
ROC curve analysis of the renal interlobar vein impedance index (RIVI) of the right kidney interlobar artery to predict preeclampsia at three different cut-points (0.3, 0.38, and 0.43).

**Figure 4.**
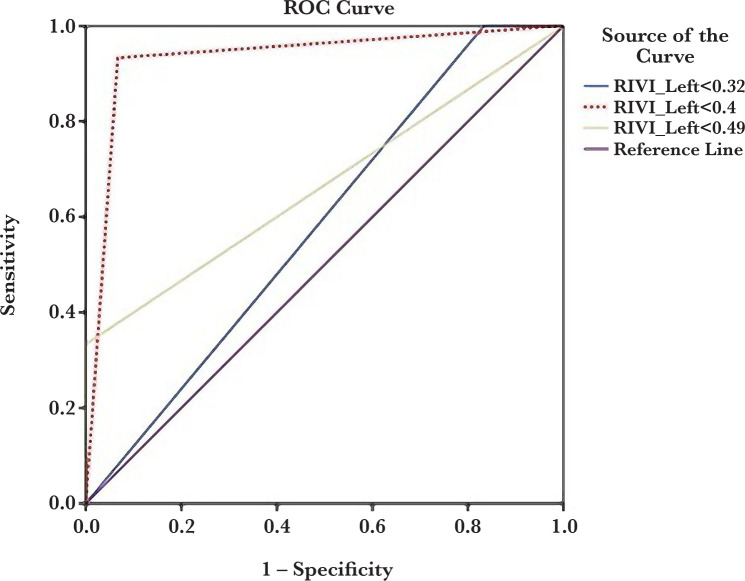
ROC curve analysis of the renal interlobar vein impedance index (RIVI) of the left kidney to predict preeclampsia at three different cut points (0.32, 0.4, and 0.49).

Table 3 shows the sensitivity, specificity, PPV and NPV of the right and left kidney RIVI values for the prediction of preeclampsia. The optimal cut-point for right and left kidney RIVI values were 0.38 and 0.4, respectively. As seen in Table 3, left kidney RIVI had better diagnostic accuracy than right kidney-related values in the prediction of preeclampsia. ROC curve analysis results for three cut-points are presented in Figures 3 and 4.

**Table 3.** Diagnostic accuracy of right and left kidney renal interlobar vein impedance index in the prediction of preeclampsia.

	Cut-point	Sensitivity	Specificity	PPV	NPV	Accuracy	AUC	P-value
**Right kidney**	0.38	66.7%	96.6%	90.9%	85.2%	86.6%	0.81	0.001
**Left kidney**	0.4	93.3%	93.3%	87.5%	96.5%	93.3%	0.93	<0.001

AUC – area under curve; NPV – negative predictive value; PPV – positive predictive value.

**Table 4.** Multiple logistic regression model to determine significant predictors of preeclampsia.

	Coefficient	SE	P-value	Odds ratio (OR)	95% confidence interval
**Right kidney RI**	-0.5	1.01	0.62	0.606	0.048 to 4.38
**Left kidney RI**	-2.37	1.28	0.06	0.093	0.008 to 1.14
**Right kidney RIVI**	0.839	1.53	0.58	2.31	0.113 to 47.28
**Left kidney RIVI**	4.26	1.54	0.006	17.14	3.46 to 47.28

### Regression model to predict preeclampsia

According to the multiple logistic regression model, only left kidney RIVI (adjusted odds ratio= 17.14, 95% CI – 3.46 to 47.28) was determined as a significant predictor of preeclampsia (Table 4).

## Discussion

Based on the results of this study, there was no significant difference regarding age, gestational age, and BMI between women with preeclampsia and women who had a normal pregnancy. Therefore, it can be concluded that these factors cannot be considered confounding factors. Based on the results of this study, there was a significant difference between the left renal interlobular artery RI and the right and left RIVI values between preeclampsia and normal pregnancy. However, there was no significant difference between the right renal arterial pressure indices in the preeclampsia and normal pregnancy groups.

The diagnostic value of right and left interlobar RI values at the 0.59 and 0.6 cut-points was significant in predicting preeclampsia. The diagnostic value of right and left kidney RIVI values were significant at cut-points of 0.38 and 0.4 in predicting preeclampsia. In a study by Bateman *et al.*, in accordance with our results, the authors concluded that there was no significant difference between renal artery RI between the preeclampsia and normal pregnancy groups [6]. In a study by Gyselares *et al.*, it was reported that RIVI was significantly higher in the preeclampsia group than in the normal pregnancy group [10]. In agreement with our findings, it was also reported that the difference was more significant regarding the left side kidney. We also observed that left renal RIVI was the only significant predictor for preeclampsia according to multiple logistic regression analysis. In the mentioned study, left kidney RIVI in the preeclampsia group was 0.44, which was higher than the left kidney RIVI seen in uncomplicated pregnancies (0.36) [10]. In another study, comparable to our findings, the authors reported that RIVI was higher in patients with early-onset preeclampsia than in late-onset preeclampsia [11]. Gyselaers *et al.* was reported that RIVI values in both right and left kidneys were higher in late-onset preeclampsia as well as early-onset preeclampsia than in normal pregnancy [9]. These values were also higher when compared to the gestational hypertension group.

Maternal venous hemodynamic disturbance has been suggested to play a role in preeclampsia for many years. In several reports by a single group of researchers, the role of raised RIVI in the prediction of preeclampsia has been verified. However, another study did not show a significant role for RIVI in the prediction of preeclampsia [3]. However, a major difference between the mentioned study and ours is that our measurements were made after the 20^th^ week of pregnancy. Nevertheless, in the study that did not report a significant role for RIVI, the measurements were made in the first trimester [3].

In the study of Lubomirova *et al.*, the authors found that there was no significant difference between the RI and PI indices in intercostal kidney arteries in pregnant women in the third trimester with preeclampsia, normal pregnant women, and non-pregnant women [12]. In a study by Bahser *et al.*, interlobar artery RI in pregnant women with preeclampsia after the 24^th^ week of pregnancy was higher than normal pregnancy groups, which is in agreement with our results [13].

However, our study had some limitations. First, given the limited number of similar studies in predicting preeclampsia in pregnant women, it is not possible to compare our study results with other studies thoroughly, and further studies should be conducted in the future. Second, the sample size was not high in this study. Therefore, there is no difference in some parameters in the present study due to the low sample size. Future studies with higher sample sizes should be designed.

## Conclusion

Based on the results of this study, it seems that the use of renal interlobar vein impedance could be reliable in predicting preeclampsia in pregnant women, along with other routine methods for the diagnosis of preeclampsia.

## Acknowledgments

This article is based on a thesis submitted to the Office of Graduate Studies in partial fulfillment of requirements for the degree of Radiology by Mohammad Saied Bazargan at the Kermanshah University of Medical Sciences, Faculty of Medicine.

### Funding

Financial support was received from the Research Council of the Kermanshah University of Medical Sciences (Grant no. 94489).

### Ethical approval

The approval for this study was obtained from the Ethics Committee of the Kermanshah University of Medical Sciences (approval ID: IR.KUMS.REC.1394.127).

### Consent to participate

Written informed consent was obtained from the participants.

### Conflict of interest

The authors declare that there is no conflict of interest.
